# Emergency Department Syphilis Screening: Insights from a National Stakeholder Convening

**DOI:** 10.1016/j.acepjo.2026.100494

**Published:** 2026-08-20

**Authors:** Kimberly A. Stanford, Elissa M. Shechter-Perkins, Douglas White, Stephen Contag, Shannon Dowler, Herbert Duber, Kiran Faryar, Jason Haukoos, Rebekah Horowitz, Katherine Hsu, Jesse Pines, Jason Zucker, Michael S. Lyons

**Affiliations:** 1 Section of Emergency Medicine, Department of Medicine, University of Chicago, Chicago, Illinois, USA; 2 Department of Emergency Medicine, Boston Medical Center, Boston, Massachusetts, USA; 3 Department of Emergency Medicine, Alameda Health System - Highland Hospital, Oakland, California, USA; 4 Department of Obstetrics, Gynecology and Women's Health, University of Minnesota, Minneapolis, Minnesota, USA; 5 Mecklenburg County Health Department, Charlotte, North Carolina, USA; 6 Department of Emergency Medicine, University of Washington, Seattle, Washington, USA; 7 Department of Emergency Medicine, University Hospitals Cleveland Medical Center, Cleveland, Ohio, USA; 8 Department of Emergency Medicine, Denver Health, Denver, Colorado, USA; 9 National Association of County & City Health Officials, Washington, DC, USA; 10 Department of Pediatrics, Boston Medical Center, Boston, Massachusetts, USA; 11 US Acute Care Solutions, Canton, Ohio, USA; 12 Division of Infectious Diseases, Columbia University, New York, New York, USA; 13 Department of Emergency Medicine, The Ohio State Medical Center, Columbus, Ohio, USA

## Abstract

The United States is experiencing a prolonged syphilis epidemic, with high rates of infection and increasing cases of congenital syphilis. Despite these trends, there is no coordinated national approach to syphilis screening outside of pregnancy, and many individuals at highest risk lack access to routine outpatient care. Emergency departments (EDs), which serve as a critical safety net for populations at highest risk of syphilis and its complications, represent an important and underutilized setting for syphilis screening. To address this gap, a national convening of multidisciplinary experts was organized by the Emergency Medicine Transmissible Infectious Diseases and Epidemics consortium, with support from the Centers for Disease Control and Prevention and the National Association of County and City Health Officials. Participants reviewed available evidence and shared implementation experience to identify feasible and effective strategies for ED-based syphilis screening. Inperson discussion was followed by iterative review and revision of manuscript drafts until consensus was achieved. A tiered framework for ED syphilis screening is proposed, including universal, population-focused, and minimum screening strategies, allowing adaptation to local resources and epidemiology. Key operational considerations include consent processes, laboratory testing approaches, electronic health record integration, and systems for follow-up and treatment. Successful implementation requires infrastructure beyond the ED, including partnerships with public health agencies, supportive policy, and sustainable financing. ED-based syphilis screening represents a critical opportunity to improve early detection, reduce transmission, and prevent congenital syphilis. Coordinated national guidance and investment are needed to support widespread adoption and maximize public health impact.

## 1 Introduction

The United States is experiencing a prolonged syphilis epidemic, with historically high rates of all stages of syphilis and a continuing rise in congenital syphilis cases.^1^ Syphilis can remain asymptomatic and difficult to detect for years, and can have dramatic consequences for individuals, families, and health systems.^2^ There is currently no robust national approach to syphilis screening outside of pregnancy. Contact tracing, whereby health department disease intervention specialists elicit reported contacts of those known to be infected, is both cost- and resource-intensive, creating additional strain on a taxed public health system. Outpatient settings, such as primary care, obstetrics, and sexual health clinics, are traditionally preferred venues for diagnosis and treatment of sexually transmitted infections (STIs); however, structural barriers, including lack of insurance, limited access to timely outpatient care, and widening inequities, have increasingly shifted this responsibility to emergency departments (EDs).

EDs are increasingly recognized as a critical location for public health interventions, including screening for epidemic infectious diseases. EDs have broad access to many populations, including those at highest risk for undiagnosed syphilis, who may not otherwise engage in routine preventive or prenatal care. As a result, routine syphilis screening in nontraditional locations, such as the ED, has been recommended by state^3^ and national agencies,^4^ alongside more established screening interventions, such as those for HIV and hepatitis C. However, although limited research demonstrates the effectiveness of ED syphilis screening,^5^, ^6^, ^7^ and national organizations, such as the American Medical Association, have recently developed toolkits^8^ that include routine ED syphilis screening, detailed and specific guidelines needed to support real-world implementation in the ED have remained elusive. Most research has focused on increasing screening among ED patients with signs or symptoms of gonorrhea or chlamydia,^9^^,^^10^ and a recent American College of Emergency Physicians quality measure ultimately excluded syphilis screening in this context, due to perceived complexity by some of the expert panelists, preventing consensus.^11^ Notably, a subsequent quality measure was adopted in 2025 supporting syphilis testing in pregnant individuals in the ED,^12^ but the scope of screening supported by this measure remains limited. It is clear that if we are to address this epidemic, a systematic strategy for screening, that goes beyond just those individuals with STI symptoms or current pregnancy, is needed. This strategy must consider effectiveness in context with feasibility, informed by experts with knowledge of the unique constraints of the ED environment as well as the complexities of syphilis diagnosis and treatment.

To address the urgent need for a coordinated and feasible approach to ED syphilis screening, a national convening of experts was organized by members of the Emergency Medicine Transmissible Infectious Diseases and Epidemics (EMTIDE) consortium, sponsored by the Centers for Disease Control and Prevention (CDC) and the National Association of County and City Health Officials (NACCHO). The objectives were to identify actionable ED syphilis screening strategies that balance effectiveness with feasibility; define the infrastructure necessary to support screening, treatment, and follow-up; identify barriers and gaps to implementation; and develop an action-oriented roadmap to advance ED-based syphilis screening nationally. The summary of the proceedings was reviewed and approved by all present, and later, recirculated to all authors to confirm continued consensus in the context of an evolving national landscape surrounding syphilis prevention and control.

## 2 EMTIDE Convening Sponsored by CDC and NACCHO

The convening was held on June 11, 2024, assembling subject matter experts in ED screening and syphilis management. Participants were invited from across the United States, selected for relevant clinical, public health, policy, and/or research expertise, and included 33 inperson attendees, 4 virtual attendees, and 1 additional contributor who participated via preconvening discussion. Attendees represented a broad range of disciplines and organizations, including 13 emergency physicians (2 of whom also represented county or state health departments), 5 additional health department leaders, 3 infectious disease physicians, and 6 representatives of payor organizations, including the Centers for Medicare and Medicaid Services (CMS). Governmental participants included representatives from the CDC, National Institutes of Health, and the Office of Infectious Disease and HIV/AIDS Policy (OIDP). Additional attendees included representatives from the Indian Health Service and leaders of major national service organizations, including NACCHO, the National Coalition of STD Directors, and the O’Neill Institute. Attendees were selected by the organizing committee for their expertise in areas relevant to syphilis or ED screening or operations, and recommendations for additional attendees were solicited from the invited experts.

### 2.1 Consensus Development

Recommendations were developed through an expert consensus process consisting of an inperson meeting, followed by iterative review and revision of successive manuscript drafts until consensus was achieved. Before the meeting, the organizing committee identified key questions related to ED syphilis screening, including strategies and tactics for developing and implementing screening programs, such as choice of screening parameters, consent, and operational barriers; system-level considerations, including regulatory, financial, and data management challenges; and clinical operations, including identification of active cases and optimal approaches to linkage to treatment. These topics formed the basis of structured discussions during the meeting, in which participants reviewed the available evidence and shared clinical experience related to syphilis and other infectious disease screening in the ED and other care settings, striving to reach consensus where possible. Discussions were informed by emerging data from national surveys, published literature, and early implementation experiences in ED syphilis screening. Following the meeting, a draft consensus statement was prepared and circulated to all participants. Recommendations were refined through iterative rounds of review and revision. Areas of disagreement were discussed by the full group and resolved through discussion and modification of the language until all participants were willing to endorse the final recommendations. All participants contributed equally to discussion and review of the recommendations; no formal weighting of opinions was applied.

### 2.2 Evidence for ED Syphilis Screening

The discussion began with a review of existing data related to ED syphilis screening. It is clear that neither broad, universal ED syphilis screening programs nor smaller-scale, risk-based or focused syphilis screening programs are common in United States EDs.^13^ Most EDs utilize diagnostic testing in patients with symptoms of syphilis, but the methods for managing positive results are variable. Despite the lack of systematic testing for syphilis in EDs, available data suggest that syphilis prevalence among ED populations is substantial and often exceeds that observed in traditional outpatient settings.^5^^,^^14^ Studies evaluating both targeted and universal screening approaches have consistently demonstrated high positivity rates,^5^^,^^15^, ^16^, ^17^ including among patients without STI-related symptoms, with one study finding a 1.7% population prevalence of untreated syphilis among ED patients undergoing nontargeted universal screening.^5^ Rates of syphilis among patients categorized as “low risk” have been comparable with those deemed “high risk.”^18^ Notably, emerging evidence indicates that a majority of syphilis cases identified through universal ED screening occur among asymptomatic individuals.^5^

Universal screening may also dramatically increase detection among pregnant patients,^5^ an outcome of particular importance for congenital syphilis prevention. Syphilis prevalence among pregnant patients without prenatal care presenting to EDs has been shown to exceed that observed among patients seen in obstetric clinics.^19^ Collectively, these findings challenge the effectiveness of narrowly targeted screening approaches and support consideration of broader screening strategies.

## 3 Screening Strategies: A Tiered Framework

There was an agreement among participants that syphilis screening in the ED is an appropriate and important public health strategy, given the realities of the current United States health system. Lessons learned from the implementation and refinement of ED HIV screening over the past 2 decades are directly relevant to the development of syphilis screening strategies. These include the impact of nontargeted screening on reducing stigma, the importance of partnerships outside the ED, and the crucial role the ED plays in reaching individuals with limited access to care.

Participants agreed that no single screening model will be appropriate for all EDs. Differences in patient populations, local syphilis prevalence, resource availability, and existing screening infrastructure necessitate a flexible, tiered framework that allows EDs to adopt strategies aligned with their readiness and capacity. However, an aggressive, strategically implemented approach could have a large impact on transmission and prevalence of syphilis over a relatively short period of time, which potentially could be followed by a more targeted approach once the epidemic has slowed. Conversely, a phased approach that begins with targeted screening and broadens once feasibility has been shown, and capacity has been expanded, may also be successful in some ED settings.

## 3.1 Three Tiers of Screening Models Were Proposed

### 3.1.1 Universal screening

The convened experts believed that universal, routine, opt-out syphilis screening offers the greatest potential to rapidly reduce transmission and prevent congenital syphilis. Universal screening simplifies implementation by eliminating the need for risk stratification, increases reach by including asymptomatic individuals, and increases screening among pregnant patients and their partners.^5^ Universal ED screening has been validated and recommended^20^ as the optimal approach for HIV, an infection that is similar in its risk factors, potentially long asymptomatic period, and associated stigma. The universal approach may be most feasible in EDs with existing opt-out HIV screening infrastructure and in geographic areas with high syphilis prevalence. This type of screening can easily be automated as part of the electronic health record (EHR).^21^ However, it generates a high volume of positive results, necessitating robust systems for follow-up and treatment that extend beyond the ED, as discussed below.

### 3.1.2 Population-focused screening

Focused or targeted screening strategies—such as screening pregnant patients, women of reproductive age, or individuals with a prior STI diagnosis—may represent a middle ground between effectiveness and feasibility. These strategies are often considered attractive because they limit resource utilization by focusing only on populations of highest interest, such as pregnant patients, who are of high urgency to diagnose given the congenital syphilis epidemic, or those presenting for STI testing, who may have the highest likelihood of syphilis infection. Indeed, most of the existing literature on syphilis screening reports results of a targeted approach.^6^^,^^7^^,^^14^^,^^17^ Such an approach may improve buy-in among staff and leadership because the motivation for screening is more intuitive, and the scale of the program feels more feasible. However, these approaches risk missed opportunities, particularly when pregnancy status is determined late in the ED visit or when partners capable of transmitting infection are not screened. Risk-based screening programs, for example, those utilizing a sexual history questionnaire, were considered by the group to be infeasible in the ED setting and likely to increase stigma while resulting in poor screening participation.^22^ Behavioral risk factors should continue to inform clinician-initiated testing for syphilis on an individual basis, however.

#### 3.1.3 Minimum strategy: comprehensive STI screening

At a minimum, syphilis testing should be included whenever other STI testing is performed in the ED. Many clinicians viewed this as standard clinical care rather than a formal screening program, though noted that even this level of screening is rare at many institutions,^23^ since it requires phlebotomy that may not otherwise be part of the patient’s care. Of note, testing for syphilis, when individuals present with symptoms of syphilis, is not considered screening, but rather diagnostic testing. Participants emphasized that this minimum strategy alone is unlikely to alter the trajectory of the syphilis epidemic meaningfully; however, it may represent a practical entry point for EDs that are reluctant to introduce broader screening initiatives. When adopted, this approach should be framed as a first step toward more comprehensive population screening as capacity allows.

Importantly, participants agreed that tiered recommendations—articulating universal, focused, and minimum strategies—would facilitate broader adoption across diverse ED settings while maintaining a clear pathway toward more impactful approaches. Additionally, any routine screening program (e.g., universal or focused) should be supplemented by additional syphilis testing at each visit for new STI symptoms, regardless of the timing of the most recent syphilis test, similar to what is recommended for HIV screening.

## 4 Operational Considerations in the ED

### 4.1 Consent and Patient Notification

There was agreement that syphilis screening, when implemented as a public health intervention, should not require consent beyond standard medical consent. Informing patients about screening should be achieved with minimal disruption to workflow, such as through posted signage or brief standardized language incorporated into routine care processes.^24^

### 4.2 Laboratory Testing

Reverse sequence testing algorithms, which initiate screening with an automated treponemal immunoassay (EIA/CIA) rather than a manual nontreponemal test (RPR), were endorsed as the preferred laboratory model for ED screening programs. The advantages of reverse sequence testing algorithms over traditional testing strategies include increased sensitivity for early or late latent infection and higher throughput for laboratories. Although point-of-care (POC) testing offers potential advantages in settings with high rates of loss to follow-up, emergency physicians expressed concern regarding added workflow burden and increased length of stay compared with laboratory-performed testing, in which results can be finalized after ED discharge. A hybrid approach was proposed, in which POC testing is reserved for populations at highest risk of adverse outcomes (e.g., pregnant patients) or loss to follow-up, whereas traditional laboratory-based testing is used for other patients.

### 4.3 EHR Integration

Automation through the EHR was identified as essential for sustainable implementation of ED-based syphilis screening. Automatic ordering, identification of individuals eligible for screening based on an institution’s screening strategy, clinical decision support tools for treatment, and shared EHR resources across institutions and departments of public health were viewed as strategies that could substantially reduce clinician and staff burden while improving consistency and reliability of screening practices.

### 4.4 Follow-Up and Treatment

Ensuring timely notification, evaluation of syphilis status, and treatment of patients with positive results and untreated syphilis emerged as the most challenging component of ED screening programs and is critical to address before a screening program can be feasibly undertaken. Complexity of follow-up was considered the major way in which syphilis screening programs would diverge from HIV screening, as the patient volume would likely be much higher and, unlike HIV, the need for treatment of syphilis cannot be confidently determined from laboratory results alone. Participants emphasized that responsibility for follow-up cannot rest solely on ED clinicians, particularly in the context of universal screening models. Effective screening programs will require partnerships with local and state health departments, disease intervention specialists (DIS), outpatient clinics, and hospital systems. “We do the test, you do the rest,” an informal model frequently cited by participants, in which the ED performs the screening but a designated partner outside the ED assumes responsibility for result review, verification of prior treatment history and syphilis status, and linkage to treatment, was considered to be particularly conducive to successful screening implementation.

The exact entity or individual responsible for result notification will depend on the availability of local resources. Proposed solutions included embedding a dedicated health department employee (DIS) in the ED, designating a pharmacist or midlevel clinician within the ED for all follow-up calls, partnering with the local health department for postvisit result notification, and partnering with a dedicated clinic within the hospital system—an approach that has been successful in at least one model.^25^ However, the group acknowledged that much of the diagnosis and treatment of syphilis will at least partially be undertaken by clinicians in the ED. A clinical guideline document for EDs implementing syphilis screening, to include streamlined guidance on managing patients with suspected or confirmed syphilis in the ED and developing follow-up infrastructure to facilitate management after the ED visit, is being separately developed.

### 4.5 Identification of Active Cases

Syphilis is uniquely difficult to diagnose, as standard laboratory testing may remain positive even after successful treatment. As a result, laboratory data alone are often insufficient to determine whether infection is active or treatment is currently indicated, making clinical history essential. Access to prior syphilis testing and treatment history was identified as a major barrier, as verification of prior treatment often requires substantial time and resource utilization. Confirmatory test results are commonly not yet available at the time of ED discharge, requiring clinicians to make management decisions in the context of incomplete data. Further, patients may be difficult to reach to confirm syphilis history after leaving the ED, and they may visit multiple hospital systems utilizing disparate EHRs.

Centralized databases, ideally maintained within public health systems and interoperable with EHRs, could streamline determination of infection status and inform management decisions. Clear, concise clinical guidance based on treatment history, clinical symptoms, and exam findings is also needed to support ED clinicians in interpreting test results and determining when empiric treatment is appropriate.

### 4.6 Empiric Treatment in the ED

Given these diagnostic limitations, participants emphasized the importance of empiric treatment during the ED visit for those with a positive initial screening test, especially for select populations in whom delayed or missed treatment carries substantial risk. These include pregnant individuals, individuals at high risk of loss to follow-up (e.g., those experiencing unstable housing, substance use disorder, or significant mental health concerns), and patients with a high likelihood of infection (e.g., those presenting with symptoms of syphilis). Treatment decisions may be guided by initial screening antibody tests or POC test results, when available. However, because laboratory testing may be negative early in the infection or result after the conclusion of the ED visit, patients with symptoms consistent with primary or secondary syphilis should receive treatment in the ED regardless of laboratory results. For populations unlikely to return for follow-up, providing treatment while the patient remains in the ED was viewed as critical to preventing missed opportunities for care and reducing downstream morbidity and transmission.

## 5 Policy and Financial Considerations

Participants emphasized that widespread adoption of ED-based syphilis screening requires department- and hospital-level support. Key to obtaining this support is the creation of explicit national recommendations and supportive policy. Endorsements from federal and state agencies, as well as professional organizations, will help normalize syphilis screening as a standard and important ED intervention, address legal concerns related to consent, and facilitate reimbursement.

Reimbursement and financial sustainability remain a central concern. Although EDs may not directly benefit financially from screening, health systems bear substantial costs associated with missed syphilis diagnoses, particularly the cases of congenital syphilis. Framing syphilis screening as a health care system-level investment with downstream cost savings was viewed as essential for gaining institutional support. Payor representatives indicated that screening programs that are aligned with national recommendations are likely to be reimbursed, underscoring the importance of clear national guidelines. Cost-effectiveness of universal HIV^26^ and hepatitis C^27^ screening in the ED has been demonstrated in multiple studies, and it was thought likely that syphilis screening would be similar, given the high potential costs associated with untreated disease. However, participants also emphasized the need for further cost-effectiveness studies evaluating the costs associated with syphilis screening programs and the downstream consequences of missed syphilis diagnoses, to inform implementation decisions and reimbursement expectations.

## 6 Summary and Conclusions

Convening participants universally agreed that ED-based syphilis screening should be recognized as a critical component of the national response to the syphilis epidemic. Reduction in all syphilis cases should be the overarching goal, but with a particular emphasis on strategies that maximize reduction of congenital syphilis. Universal screening, supplemented by targeted approaches and episodic symptom-driven testing, was endorsed by the participants as having the greatest potential for rapid impact, particularly in high-prevalence settings, whereas population-focused and symptom-driven screening protocols are important options for communities with lower disease prevalence or other barriers to implementing large-scale screening programs.

EDs with opt-out HIV screening already in place may be best placed to implement universal syphilis screening, as much of the existing infrastructure can be easily adapted. Although many EDs may not be prepared to adopt universal screening immediately, some may be willing to implement more limited screening strategies as an initial step. In such settings, the goal should be to progress toward broader screening approaches as capacity allows, and in line with local epidemiology. Given that syphilis is a curable disease, an aggressive approach implemented over a defined period could potentially alter the trajectory of the epidemic, after which more targeted strategies may be appropriate.

The success of any ED-based syphilis screening program depends on coordinated infrastructure outside the ED for follow-up and treatment, strong partnerships with public health agencies, supportive policy, and sustainable financing. State and national agencies should provide clear guidance regarding the role of screening in the ED, which in turn could facilitate reimbursement. Future research should focus on identifying optimal screening strategies and conducting financial analyses to better characterize health system costs or savings associated with both the screening programs themselves and potential missed syphilis diagnoses in the absence of screening.

EDs have repeatedly demonstrated their capacity to respond to public health crises; with appropriate support and guidance, ED-based syphilis screening can play a transformative role in reversing current trends and preventing future cases of congenital syphilis. This moment presents an opportunity to act decisively, leveraging the unique position of the ED to reach those most at risk and to advance equity in infectious disease prevention.

## Funding and Support

By *JACEP Open* policy, all authors are required to disclose any and all commercial, financial, and other relationships in any way related to the subject of this article as per ICMJE conflict of interest guidelines (see www.icmje.org).This work was funded by a grant from the 10.13039/100000030Centers for Disease Control and Prevention and the 10.13039/100021816National Association of County and City Health Officials.

## Author Contributions

All authors participated in generating the content of this commentary. KAS drafted the manuscript, and all authors assisted with editing the manuscript.

## Conflict of Interest

All authors have affirmed they have no conflicts of interest to declare.
