# Supplementary material for: Emergency Department Syphilis Screening: Insights from a National Stakeholder Convening
Source: J Am Coll Emerg Physicians Open. 2026 Aug 20;7(5):100494. doi: 10.1016/j.acepjo.2026.100494 (PMC13522367; doi:10.1016/j.acepjo.2026.100494)
Supplement: Supplementary file 1 [file mmc1.docx]

| Appendix 1: Convening Attendees | | |
| --- | --- | --- |
| **Government/Non-governmental Organizations** | | |
|  | Lindley Barbee, MD, MPH | Centers for Disease Control and Prevention (CDC) |
|  | Jennifer Ludovic, DrPH | Centers for Disease Control and Prevention (CDC) |
|  | Robert McDonald, MD, MPH | Centers for Disease Control and Prevention (CDC) |
|  | Travis Austin, MD, MPH | Indian Health Service (IHS) |
|  | Loretta Craig, MSN, FNP-C | Indian Health Service (IHS) |
|  | Rebekah Horowitz, JD, MPH | National Association of County and City Health Officials (NACCHO) |
|  | Rachel Deitch | National Coalition of STD Directors (NCSD) |
|  | Carolyn Deal, PhD* | National Institute of Allergy and Infectious Diseases (NIAID) |
|  | Jeffrey Levi, PhD | George Washington University |
|  | Shannon Dowler, MD, CPE | Mecklenburg County Health Department Office of the Assistant Secretary for Health (OASH) |
|  | Babak Yaghmaei, MPH | Office of Infectious Disease and HIV/AIDS Policy (OIDP) |
|  | Sonia Canzater, JD, MPH | O’Neill Institute for National and Global Health Law |
| **Health Departments** | | |
|  | Kathleen Jacobson, MD | California Department of Public Health |
|  | David Kern | Chicago Department of Public Health |
|  | Irina Tabidze, MD | Chicago Department of Public Health |
|  | Kristopher Lyon, MD^†^ | Kern County Department of Public Health |
|  | Thomas Dobbs, MD | Mississippi State Department of Health University of Mississippi |
|  | Herbie Duber, MD, MPH^†^ | Washington State Department of Health University of Washington |
| **Emergency Physicians** | | |
|  | Douglas White, MD | Alameda Health System/Highland Hospital |
|  | Elissa Schechter-Perkins, MD, MPH, DTMH | Boston Medical Center |
|  | Jason Haukoos, MD, MS | Denver Health |
|  | Richard Rothman, MD, PhD | Johns Hopkins University |
|  | Michael S. Lyons, MD, MPH | The Ohio State Medical Center |
|  | Phillip Moschella, MD, PhD | PRISMA Health/USCSOM Greenville |
|  | Kimberly Stanford, MD, MPH | University of Chicago |
|  | Kiran Faryar, MD, MPH | University Hospitals Cleveland Medical Center |
|  | Jesse Pines, MD, MBA | US Acute Care Solutions |
| **STI Experts** | | |
|  | Kathy Hsu, MD, MPH | Boston Medical Center Massachusetts Department of Public Health Ratelle Prevention Training Center |
|  | Jason Zucker, MD | Columbia University New York City Prevention Training Center |
|  | Aniruddha Hazra, MD | University of Chicago |
|  | Stephen Contag, MD | University of Minnesota |
| **Financial Experts** | | |
|  | Jasmine Dhindsa, MD*^,‡^ | Centers for Medicare & Medicaid Services |
|  | Jessica Lee, MD, MSH*^,‡^ | Centers for Medicare & Medicaid Services |
|  | Sean Michael, MD, MBA, FACEP^†,‡^ | Centers for Medicare & Medicaid Services |
|  | Tanya Raggio, MD, MPH*^,‡^ | Centers for Medicare & Medicaid Services |
|  | Yolanda Lawson, MD | Health Care Services Corporation National Medical Association Baylor University Medical Center |
|  | Douglas Fish, MD | Office of Health Insurance Programs New York State Department of Health |
|  | Thomas Spiegel, MD, MBA^†^ | Chief Quality Officer, University of Chicago |

*Virtual Attendee Only

†Also an emergency physician

‡Participated via pre-convening discussion
